# A Public Mid-Density Genotyping Platform for Hexaploid Sweetpotato (*Ipomoea batatas* [L.] Lam)

**DOI:** 10.3390/genes15081047

**Published:** 2024-08-09

**Authors:** Dongyan Zhao, Alexander M. Sandercock, Maria Katherine Mejia-Guerra, Marcelo Mollinari, Kasia Heller-Uszynska, Phillip A. Wadl, Seymour A. Webster, Craig T. Beil, Moira J. Sheehan

**Affiliations:** 1 Breeding Insight, Cornell University, 525 Tower Rd, Ithaca, NY 14853, USA; dz359@cornell.edu (D.Z.); ams866@cornell.edu (A.M.S.); mariakmejia@gmail.com (M.K.M.-G.); ctb94@cornell.edu (C.T.B.); 2 Bioinformatics Research Center, North Carolina State University, Campus Box 7609, Raleigh, NC 27695, USA; mmollin@ncsu.edu; 3 Diversity Arrays Technology, Canberra, ACT 2617, Australia; kasia@diversityarrays.com; 4 US Vegetable Laboratory, United States Department of Agriculture, Agricultural Research Service, Charleston, SC 24914, USA; phillip.wadl@usda.gov; 5 Department of Plant, Soil Sciences and Engineering, College of Agriculture, Science, and Education (CASE), Port Antonio P.O. Box 170, Portland, Jamaica; seymour.webster@case.edu.jm

**Keywords:** sweetpotato, amplicon-sequencing, plant breeding, DArTag genotyping, microhaplotype

## Abstract

Small public breeding programs focused on specialty crops have many barriers to adopting technology, particularly creating and using genetic marker panels for genomic-based decisions in selection. Here, we report the creation of a DArTag panel of 3120 loci distributed across the sweetpotato (*Ipomoea batatas* [L.] Lam) genome for molecular-marker-assisted breeding and genomic prediction. The creation of this marker panel has the potential to bring cost-effective and rapid genotyping capabilities to sweetpotato breeding programs worldwide. The open access provided by this platform will allow the genetic datasets generated on the marker panel to be compared and joined across projects, institutions, and countries. This genotyping resource has the power to make routine genotyping a reality for any breeder of sweetpotato.

## 1. Introduction

Molecular breeding has advanced agricultural breeding programs, particularly in key staple crops such as tomatoes, corn, and barley, through techniques such as linkage mapping and marker-assisted selection [1,2,3,4]. Genomic selection and genomic prediction allow breeding for complex characteristics while reducing the time and costs associated with developing new cultivars [5,6,7]. While these advancements have directly improved the breeding programs of common crop species, their applications in specialty crops and animal breeding remain largely untapped due to a lack of comprehensive genomic resources [8]. The creation of such resources will enable precise modification of genetic traits, leading to enhanced resistance to diseases and pests, improved nutritional profiles, and better adaptability to climate change.

Sweetpotato (*I. b*) is a dicotyledonous vine that produces nutritious storage roots with wide phenotypic diversity in terms of flesh and skin color, dry matter, and perceived sweetness. Native to the tropical regions of Central and South America, sweetpotato is an essential crop worldwide, and its economic importance stems from its agricultural contribution, nutritional value, diverse uses, and adaptability. In 2020, the global sweetpotato market value was approximately USD 42.75 billion, with projections indicating that the value will rise to about USD 45.71 billion by 2025 (https://www.statista.com/statistics/1100004/global-sweet-potato-market-size/, accessed on 31 October 2023). Notably, the popularity of sweetpotato has grown significantly in the United States (US), witnessing a consumption surge of nearly 42% between 2000 and 2016 (National Statistics for Sweetpotatoes). In 2022, the value of US sweetpotato production reached USD 598.4 million (National Statistics for Sweetpotatoes).

Exotic and native pests and pathogens pose significant threats to sweetpotato cultivation, reducing overall yield and the quality of storage roots harvested by growers. These detrimental agents encompass a range of organisms, including root-knot nematodes (*Meloidogyne enterolobii* and *Meloidogyne incognita*), the sweetpotato weevil (*Cylas formicarius*), the wireworm–*Diabrotica–Systena* complex (*Agrypnus* spp., *Conoderus* spp., *Diabrotica* spp., *Gonocephalum* spp., *Melanotus* spp., and *Systena* spp.), sweetpotato flea beetle (*Chaetocnema confinis*), and white grubs (*Phyllophaga* spp.) [8,9,10]; pathogens such as Sweetpotato Leaf Curl Virus (SPLCV), transmitted by the whitefly (*Bemisia tabaci*), have the potential to cause yield reductions of up to 80% [11]. Impacts from weed interference, e.g., palmer amaranth, can reduce yield by up to 81% [12].

At present, the management of these pests primarily relies on the application of insecticides, herbicides, behavior-modifying chemicals/sex attractants, and the utilization of resistant sweetpotato cultivars. The accessibility and cost-effectiveness of pesticides can be problematic, particularly for growers in developing nations [13]. Moreover, many control measures face challenges targeting pests below the soil surface. As such, additional breeding efforts to develop novel pest-resistant sweetpotato lines and identify resistance within existing sweetpotato germplasm are high priorities for breeders and producers. Other breeding targets include improving hardiness against suboptimal weather conditions and traits necessary to the food industry, such as color, taste, texture, cooking quality, shelf-life, and nutritional composition. 

Sweetpotato genetics presents several challenges due to its unique characteristics, including being a hexaploid (2*n* = 6x = 90), with its exact origin (autopolyploid or allopolyploid) unclear [14], having a large genome (~1.6 Gb) with a high degree of heterozygosity, and is primarily outcrossing [15]. Breeding for sweetpotato usually involves polycrossing in isolation, where only the female parent of a true botanical seed is known, and the male parent could be any other individual in the isolation block. Additionally, sweetpotato flowering is latitude-dependent and limited to the tropics, such that it does not occur naturally in latitudes higher than 35N or S [16]. Inducing flowering time in temperate growing regions is generally achieved by a short-day photoperiod combined with top-grafting with Ipomoea species (*Ipomoea nil* or *Ipomoea alba*) that do not develop storage roots [16]. Unlike many annual species, sweetpotato cultivars are distributed as clones (vegetative slips or tubers) to maintain phenotypic consistency, which bypasses the low seed production (4–6 seeds) per flower and lack of homozygosity in the genome. Despite these breeding challenges, advances in genotyping technologies have led to chromosome-level genome assemblies of the sweetpotato genome, thereby enhancing our ability to discern the genetic basis of traits of interest [17]. Contemporary crop breeding requires the integration of multiple-omics resources and multidisciplinary collaboration. Leveraging these genomic and phenomic resources can accelerate sweetpotato breeding programs by identifying marker-trait associations and genomic selection methods, reducing breeding time and costs.

Breeders hoping to adopt genomic data and methods into their programs typically find that creating a rapid genotyping pipeline is a logical place to start. However, there are several considerations they must address when selecting a genotyping platform: how will it fit within both their breeding and selection cycles, and can it deliver on their objectives? Additionally, many breeders prefer to adopt a complete workflow beginning with DNA extraction and genotyping on a platform suited to their material by a commercial vendor(s), followed by the availability of user-friendly bioinformatic tools to transform their returned raw data into a usable format to make selection decisions on time. In choosing a genetic marker platform for sweetpotato, we considered several factors: the cost per data point, vendor availability and service offerings, data turnaround times, and resulting data that breeders can utilize for a wide array of genetic analyses for their decision support. For sweetpotato, we determined that a targeted-amplicon sequenced-based approach would be the most beneficial for breeders. Compared to Genotyping-by-Sequencing (GBS), targeted, amplicon-based genotyping technologies such as DArTag (Diversity Array Technology—DArT) and Capture-Seq (LGC Genomics) have low missing data rates and query the same loci in all samples across genotyping projects, allowing new data to be easily appended to existing data [18,19,20]. The amount of data returned is in the tens of thousands or less, rather than the millions of reads (compared to GBS or WGS), simplifying downstream bioinformatics processing [19,21]. Simplified raw data processing speeds up analysis time for key selection decision actions like marker-assisted selection (MAS), introgression tracking, linkage mapping, GWAS, and genomic prediction [19]. 

Here, we report the creation of a DArTag panel of 3120 loci distributed across the sweetpotato genome. DArTag is a hybridization/amplicon-based targeted genotyping platform developed by DArT [22] (https://www.diversityarrays.com/services/targeted-genotying/, accessed on 31 October 2023). Oligos are custom designed to target known genetic variants (SNPs and InDels less than 50 bp) with their flanking genomic regions, resulting in sequencing products of 81 bp in length. To develop and validate this panel, we (1) sequenced a global sweetpotato diversity panel of 47 unique lines using whole-genome skim sequencing and selected ~3K quality markers; (2) sequenced a reciprocal sweetpotato bi-parental population and elite lines using this sweetpotato 3K DArTag panel for validation; and (3) successfully generated a linkage map using the 3K DArTag sequencing data. This 3K DArTag panel provides a novel genetic resource to the sweetpotato breeding community, offering a valuable tool for molecular breeding and genomic prediction applications. 

## 2. Materials and Methods

### 2.1. Germplasm Selection and Whole-Genome Sequencing of a Sweetpotato Diversity Panel

The authors selected a diversity panel of 47 hexaploid sweetpotato (*I. b.*) parental and founding lines from 7 regional groups (Africa, Caribbean, Far East, Pacific Islands, North America, Central America, and South America) for whole-genome skim sequencing (Table S1, “Y” in the “SNP discovery” column). Sweetpotato is not bred at the diploid level, and there are no known diploid *I. b.* accessions that are relevant to the breeding germplasm pool. Leaf tissue samples for two biological replicates of each sample in the discovery panel were prepared by Dr. Wadl and processed for DNA at Genomics Facility of Cornell Institute of Biotechnology. Sequencing libraries (average insert DNA size of 300 bp) were prepared using either Illumina’s Nextera WGS library prep at the Genomics Facility of Cornell Institute of Biotechnology or the NEBNext Ultra DNA Library Prep Kit at Novogene (Davis, CA, USA). Whole-genome sequencing with 150 bp paired-end reads was generated using an Illumina NovaSeq 6000 at Novogene (https://en.novogene.com, accessed on 1 February 2021).

### 2.2. SNP Discovery and Selection of 3K Marker Loci for Building a DArTag Genotyping Panel

**Figure 1:**
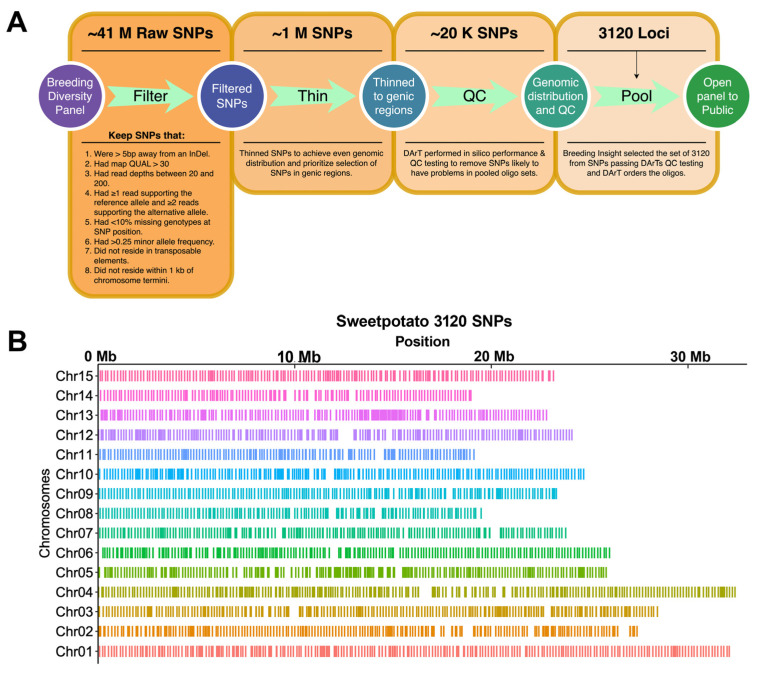
Selection of the 3120 SNPs that comprise the 3K DArTag panel. (**A**) The SNP filters and criteria applied to select the 3K DArTag panel from the WGS of a sweetpotato diversity panel. Abbreviations: M is millions, K is thousands. (**B**) The distribution of the 3120 DArTag panel SNPs across the sweetpotato physical genome.

Breeding Insight processed raw FASTQ sequences by removing residual adapter sequences and low-quality bases using Trimmomatic (LEADING:10 TRAILING:10 SLIDINGWINDOW:4:15 MINLEN:30) [23]. Cleaned reads were then aligned to the *Ipomea trifida* reference genome [15] using BWA-MEM [24]. Structural variants (SNPs and indels) were called using the DNAseq pipeline developed by Sentieon (https://www.sentieon.com, accessed on 13 July 2022) [25]. A total of ~41 M (41,625,649) SNPs were discovered from the whole-genome re-sequencing of the diversity panel, where a high-confidence set of ~1 M (931,745) SNPs (Figure 1) were obtained by requiring SNPs to (1) not be located within five bp distance to an indel; (2) have QUAL > 30; (3) have minimum and maximum read depths of 20 and 200, respectively; (4) for each sample, have at least one read supporting reference allele, and two reads supporting the alternative allele; (5) have less than 10% missing genotype per SNP position; (6) have a minor allele frequency greater than 0.25; (7) not be located in transposable elements and within 1 Kb of chromosome termini. Further thinning of the 1 M SNPs to achieve even genomic distribution (based on physical distance) and to maximize SNPs in genic regions produced a set of ~20 K (20,559) SNPs. The 20 K SNPs were submitted for QC to DArT (Diversity Arrays Technology Pty Ltd., Canberra, Australia), from which Breeding Insight selected a set of 3K (*n* = 3120) SNPs (File S1). In addition to the previous filters, the 3K SNPs were primarily selected from those with even genome distribution and locations in genic regions. Oligos were designed at DArT and synthesized by Integrated DNA Technologies (IDT, Coralville, IA, USA). 

### 2.3. Principles and Implementation of the DArTag Genotyping Assay

The DArTag genotyping assay consists of four steps based on principles described in Krishnakumar et al., 2008 [26], and implemented as described previously [27,28]. Briefly, the pool of 3120 oligos, each targeting one genetic variant plus adjacent flanking sequence, is hybridized to denatured gDNA in step 1, followed by SNP/INDEL copying into DArTag molecules by DNA polymerase in step 2. After ligation into circular molecules in step 2, nucleases treatment to remove un-circularized molecules in step 3, DArTag products are subsequently amplified in step 4 with the simultaneous addition of sample unique barcode used downstream for demultiplexing. The products of the DArTag assay, after purification and quantification, are sequenced on NGS platforms (e.g., NovaSeq 6000, X series sequencers, Illumina) with a depth of around 200×, QCed, demultiplexed, and the genetic variants are detected using the DArT proprietary analytical pipeline.

**Figure 2:**
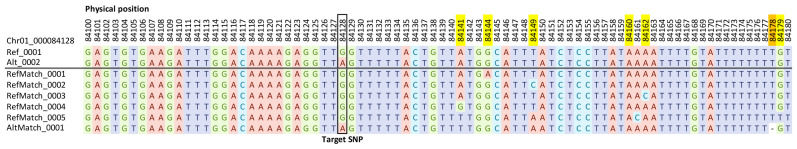
DArTag sequencing reads from locus Chr01_000084128. Each sequence is a microhaplotype detected in breeding material tested on the panel. The DArTag assay was designed to detect the target locus (box) and distinguish the Reference allele from the Alternative allele. Additional variant nucleotide positions (yellow fill) distinguish the individual microhaplotypes. An indel is indicated by “-” at the genomic position (orange fill). The top row refers to the physical nucleotide position within the genome on chromosome 1.

The sweetpotato DArTag panel produces 81 bp reads, which we used to call SNPs, or, in the case of complex genomes like sweetpotato, we used to identify microhaplotypes (Figure 2). Sequencing reads can contain variants in addition to the target SNP, allowing for the detection of more than two alleles at each of the 3120 loci. As the amplicons are very short, variants found within these reads are assumed to be in complete linkage and, therefore, can be used for improved phasing of genotyping calls and determining allele dosage. The practical maximum number of probes on a DArTag panel is 7 K loci. However, the optimal max may differ by species, genome complexity, and read depth required to sufficiently call genotypes (Andrzej Kilian, DArT, personal communication). 

### 2.4. Validation of the Sweetpotato DArTag Panel

The sweetpotato 3K DArTag marker panel was validated only on *I. b.* accessions important for cultivar breeding. We used a reciprocal bi-parental population between ‘Beauregard’ and ‘Regal’ (36 and 56 progeny, respectively), a bi-parental population between ‘Beauregard’ and ‘Uplifter’ (20 progeny), and a diverse set of elite lines of cultivated hexaploid sweetpotato (*n* = 264) (Table S1, “Y” in “Validation set” column). Tissues were grown and collected by Drs. Wadl and Webster and submitted to DArT for DNA extraction and genotyping. The raw DArTag genotyping data included FASTQ and the missing allele discovery count (MADC) file (File S2).

The MADC file was first filtered at the microhaplotype level. We retained a microhaplotype if it was present in at least ten samples, and each sample had at least two reads detected. First, samples with ≥95% missing data were removed. Then, filtering at the marker loci was based on ≥10 samples, with each having ≥10 reads for each marker locus per sample. All SNPs, including target and off-target SNPs, were extracted from all remaining marker loci for downstream analyses. Principal component analysis was conducted in R (v4.2.1; R Core Team 2022) [29] using read count data from all samples using the *AddPCA* function in polyRAD (v2.0) [30] and plotted using ggplot2 (v3.4.4) [31]. The R package updog (v2.1.5) [32] generated dosage calls using the read count data.

### 2.5. Genetic Map Construction

We generated a genetic map to exhibit the quality of the sweetpotato 3K DArTag marker placement in genetic space (since our marker selection was based on even distribution across physical space) and to provide an additional genetic tool for sweetpotato breeders. In the dataset, there were three bi-parental populations: (1) ‘Beauregard’ × ‘Regal’ (BR), its reciprocal cross, (2) ‘Regal’ × ‘Beauregard’ (RB), and (3) ‘Beauregard’ × ‘Uplifter’ (BU). We specifically focused on the BR and RB populations for map construction purposes. 

We began marker filtering by excluding markers with over 20% missing data from the entire dataset to ensure data quality and accuracy, which reduced the total number of SNPs from 3120 to 3072. We did not discard any SNPs due to low minor allele frequency (MAF). Next, we extracted polymorphic SNPs for the BR and RB bi-parental reciprocal crosses but removed any SNP missing parental information, reducing the number of SNPs from 3072 to 2410. Following this, we removed SNPs that were homozygous in both parents, resulting in 1674 SNPs. We further eliminated SNPs with redundant linkage information, which decreased the total to 1660, and a final exclusion of SNPs with more than 10% missing data culminated in a set of 1476 high-quality SNPs.

Following quality filtering, we used the software MAPpoly (v0.3.3) [33,34] to build the sweetpotato genetic map (Scripts and parameters used can be accessed here https://github.com/mmollina/sweetpotato_mid_density_genotyping_platform, accessed on 15 April 2024. A subsequent Mendelian segregation fit screening meticulously refined the number of SNPs from 1467 down to 1285 SNPs. Following this filtering, the “AGHmatrix” package in R was employed to compute the genomic relationship matrix based on the VanRaden method [35]. The derived matrix was used to perform a principal component analysis (PCA) to extract the main components of variance in the genomic data. Simultaneously, an evaluation of the individuals’ positions on the PCA plot led to the exclusion of those not falling between ‘Beauregard’ and ‘Regal’ as would be expected for true F1 progeny, reducing the initial 92 individuals down to 60 for the linkage map construction using the function “filter_individuals”. SNP markers were grouped and ordered within linkage groups based on a framework map from Mollinari et al., 2020 [34], leveraging ‘Beauregard’ as a parent in that study (Figure S1). For each linkage group, markers were phased, and maps were estimated using the multilocus algorithms implemented in MAPpoly’s “est_rf_hmm_sequential function”. The map was re-estimated utilizing the “est_full_hmm_with_global_error” function, assuming a 0.1 global error rate. 

## 3. Results

### 3.1. The Sweetpotato 3K DArTag Genotyping Panel 

We discovered ~41 M (41,625,649) SNPs from the whole-genome re-sequencing of the sweetpotato diversity panel (Figure 1). After multiple rounds of quality filtering, ~1 M (931,745) SNPs remained evenly distributed across the physical genome and enriched for SNPs residing in genes. Breeding Insight submitted ~20 K (20,599) SNPs to DArT for quality assessment, from which we selected a final set of 3120 loci containing 3120 target SNPs to create the 3K DArTag panel. Based on the closest reference genome available at the time, the diploid species *I. t.*, which is a putative progenitor of hexaploid *I. b.* [15], we designed 1501 and 1619 marker loci (File S1) to produce amplicons from the plus and minus strands, respectively. According to the gene annotation v1.0 of *I. t.*, 99% (*n* = 3091) of loci reside in genic regions, with only 1% (*n* = 29) in non-genic regions (File S1). Among the 3120 loci selected, each chromosome harbors between 137 loci on Chr14 and 308 loci on Chr05, with an average of 208 loci per chromosome. Furthermore, a positive correlation (*R^2^* = 0.58) between the number of genes on a chromosome and the number of DArTag targeted loci indicates that chromosomes with more genes have better marker coverage (Table S2). 

### 3.2. Microhaplotypes from the Sweetpotato 3K DArTag Genotyping Panel

As mentioned in Zhao et al., 2023 [27], DArTag generates genotyping results in several formats, among which the MADC format (missing allele discovery count) provides all the 81 bp microhaplotypes discovered based on amplicons for the 3K marker loci. These microhaplotypes contain target SNPs per assay design as well as off-target SNPs. To better distinguish these microhaplotypes, those matching the reference and alternative alleles at the target SNP site and containing no other variant nucleotide are denoted as Ref and Alt microhaplotypes, respectively. Additional microhaplotypes that contain off-target SNPs are denoted as RefMatch (when target SNP matches Ref) and AltMatch (target SNP matches Alt), with consecutive numbering for uniqueness (Figure 2).

To evaluate the 3K DArTag panel’s quality and applicability, we genotyped on a validation set of 376 samples, which included three bi-parental populations, and 264 elite lines utilized in the North American sweetpotato breeding programs. With the MADC report (File S2), RefMatch or AltMatch microhaplotypes were retained and assigned fixed allele IDs based on the following criteria: (1) presence in at least 5% of total samples, with each sample having a minimum of two reads; and (2) achieving a sequence alignment of ≥90% and identity of ≥90%. Of the 18,105 RefMatch and AltMatch microhaplotypes, 13,415 remained, resulting in an average increase of 4.3 alleles per marker locus. 

Next, we assessed the genetic diversity of the validation samples by calculating the number of unique microhaplotypes per marker locus. Microhaplotypes that were not present in at least ten samples were excluded, with each sample having a minimum of two reads per microhaplotype (Table S3). We categorized 94 marker loci as low-performing since they produced 0 microhaplotypes. Similarly, we identified 207 marker loci as monomorphic because they produced a single microhaplotype. Lastly, we identified 183 marker loci that produced >10 microhaplotypes, indicating high genetic diversity or potential off-target amplifications from paralogous sequences. 

### 3.3. Panel Effectiveness in Extant Sweetpotato Accessions

We evaluated the marker loci detection rate at both sample and marker levels. We removed 18 samples with >95% missing data from further downstream analyses (Table S4). We hypothesize that the high missing data of these 18 samples is most likely due to poor DNA quality because other accessions belonging to the same family or geographical regions showed low missing data. Out of the remaining 358 samples, >95% (*n* = 341) and >77% (*n* = 276) have data from ≥80% and ≥90% marker loci, respectively, indicating the high detection efficiency of the marker panel.

At the marker level, 108 (3%) out of the 3120 marker loci had signals from less than ten samples (Table S5), representing markers with low detection rates in the samples included in the genotyping project. We found that 2211 (71%) marker loci were detected in ≥95% of samples, and 164 (5%) marker loci were detected in all the samples surveyed, representing the most conserved marker loci in the sweetpotato genome and its related species.

The average missing data for ‘Beauregard’ and ‘Regal’ reciprocal cross populations was 10% (with a standard deviation of 8%), and for ‘Beauregard’ and ‘Uplifter’, the bi-parental population was 16% (with a standard deviation of 17%), where the higher missing rate for the latter was expected because Uplifter is a regional cultivar in Jamaica and was not included in the initial sweetpotato panel for marker design. The average missing data for other sweetpotato cultivars was 12%, with a standard deviation of 11% (Figure S2).

### 3.4. Creation of a Linkage Map

**Figure 3:**
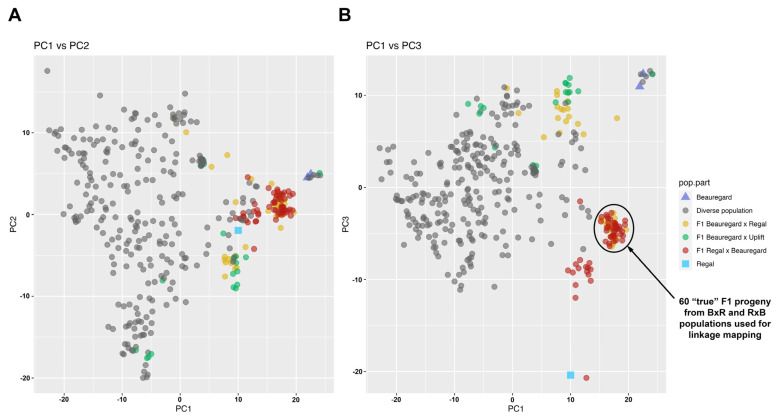
Assessment of a mid-density DArTag genotyping panel for sweetpotato using a diverse population and three bi-parental populations. (**A**) Plot of the first two principal components. (**B**) Plot of principal components 1 and 3. Each point represents an individual, colorized by germplasm passport information. The three bi-parental populations mostly cluster together, demonstrating the relatedness of individuals within these groups. However, there are some exceptions where individuals from these populations spread into the area primarily occupied by the diverse population. These patterns support the expected clustering of related individuals and scattering of unrelated ones.

The principal component analysis (PCA) revealed that the first three PC dimensions accounted for 68.9% of the total genetic variation detected within the samples (Figure 3). Related individuals, particularly full siblings, formed easily distinguishable clusters within the PCA plots, highlighting their genomic similarities. Most full-sib individuals from ‘Regal’ × ‘Beauregard’ localize between the two parents in the PCA plot (Figure 3B). Although individuals on the ‘Beauregard’ × ‘Regal’ population shared this intermediary location, 18 individuals co-localized within the diverse population. The low number of true F1 progeny in each bi-parental cross is not unexpected as inducing flowering is labor-intensive, and each manual cross produces only 4–6 seeds. The diverse population, which lacked a specific crossing design, was dispersed widely across the PCA plot, demonstrating their varied genomic diversity.

**Figure 4:**
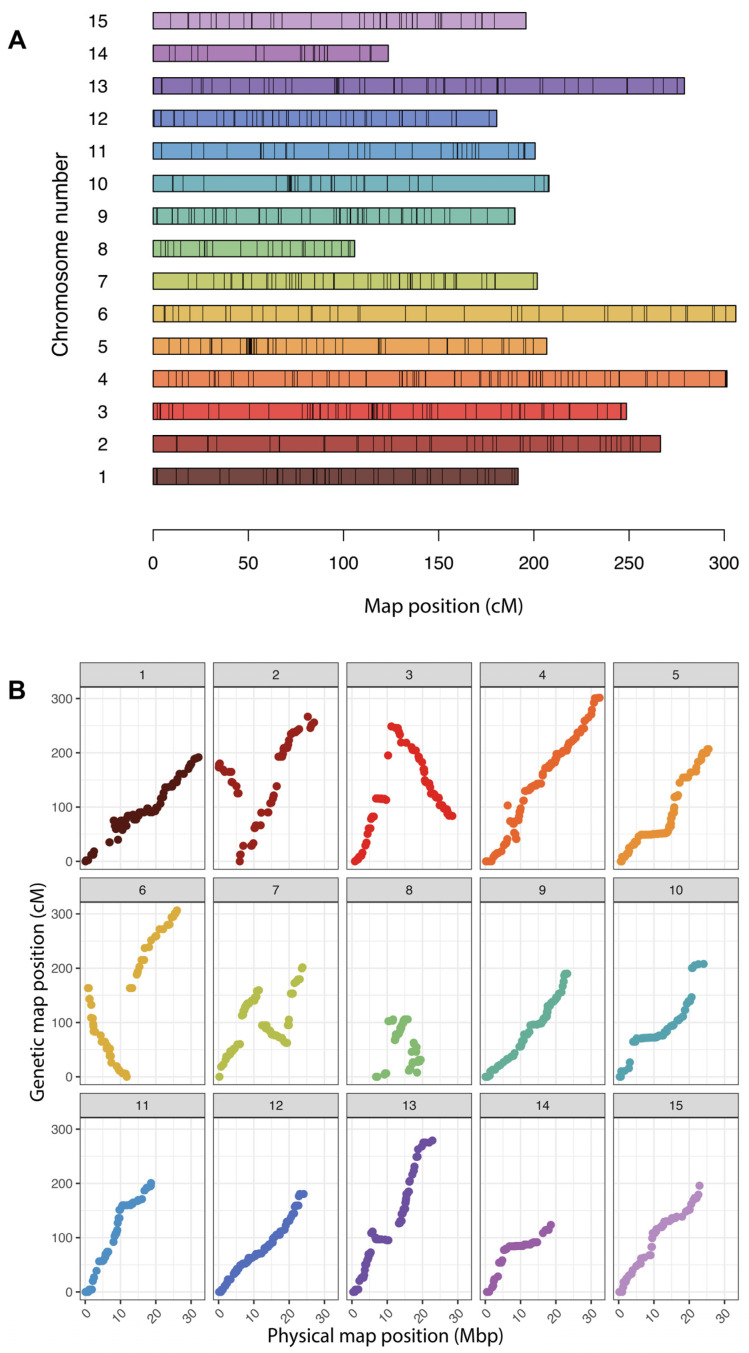
Genetic map of bi-parental reciprocal cross populations between ‘Beauregard’ and ‘Regal’. (**A**) Regeneration of the 15 linkage groups of the sweetpotato genome. The scale bar shown is in centiMorgans (cM). (**B**) Relationship plots of genetic distance (cM) to *I. t.* reference genome physical distance (Mbp) for each of the 15 linkage groups.

We filtered out monomorphic SNPs and those with more than 10% missing data. We also removed individuals not falling between the ‘Beauregard’ and ‘Regal’ from the PCA plot. We generated a linkage map for ‘Beauregard’ and ‘Regal’ populations with 60 “true” F_1_ progeny. The resulting genetic map comprised 15 linkage groups (LGs) with a total map length of 3224.13 cM (Figure 4). The average markers per centimorgan (cM) across these LGs is 0.35, and the entire map contains 101 simplex, 107 double-simplex, and 884 multiplex markers, aggregating to 1092 markers in total. In refining these linkage groups, we identified chromosomes 4 and 8 had noticeable starting gaps. We removed one marker from chromosome 4 and three markers from chromosome 8 to reduce these gaps. In contrast, chromosome 10 displayed a significant gap of 53.77 cM. However, no markers were removed from this chromosome, as it would have required eliminating 12 markers from both its extremities, all of which were interconnected.

## 4. Discussion

### 4.1. Assessment of DArTag Performance and Utility in a Complex Polyploid Genome

The DArTag platform performed better than initially expected, as sweetpotato has the largest and most complex genome for which we have created a panel. With only 60 “true” F_1_s and only 1092 markers, the genetic map we constructed was inflated (3224.13 cM total map length). The construction of the genetic map faced challenges, primarily due to two factors. Firstly, hexaploid sweetpotato datasets typically exhibit a low signal-to-noise ratio. Secondly, the full-sib population we utilized for the map construction was limited in size (*n* = 60). A larger bi-parental population should result in more accurate linkage maps with less inflation. 

In the future, breeders that employ polycross strategies in their breeding program can sample all parents and their progeny in the block and genotype using the 3K DArTag panel. From these data, breeders can determine the male parentage of each progeny and make interconnected maps by leveraging the common parents. One such population is being developed by breeders at North Carolina State University (C. Yencho and M. Mollinari, personal communications). Additional recombinant information from larger maps, along with the multi-allelic DArTag data, can be used to perform GWAS, MAS, and QTL mapping with more precision. It is too early to say if genomic selection can be implemented in sweetpotato and, if so, how many markers are needed for accurate predictions. As the community adopts molecular techniques into breeding programs, including the 3K DArTag panel, additional needs for GS can be better assessed and produced accordingly.

Since our linkage marker ordering directly utilized the framework map established by Mollinari et al., 2020 [34], the inversions and unique arrangements we identified in the physical map align with those previously reported in their comprehensive study. In essence, the rearrangements observed in our study were anticipated and agreed with the marker order delineated in the 2020 map. Those rearrangements are most likely differences between the cultivated hexaploid sweetpotato and its diploid ancestor, *I. t.*, or possibly mis-assemblies in the *I. t*. reference genome. 

A total of 183 marker loci had a high number of alleles detected (>10 microhaplotypes), indicating high genetic diversity or potential off-target amplifications from paralogous sequences. Distinguishing true on-target alleles from off-target ones will be our focus in another study when a more comprehensive sample panel is genotyped. Before that, it is highly recommended that only the Ref and Alt microhaplotypes from these highly polymorphic marker loci be used in analyses.

In contrast, 108 marker loci out of the 3120 marker loci had signals from less than ten samples (Table S5), representing markers with low detection rates in the samples tested. Also, 207 marker loci produced a single allele (monomorphic) in the validation test. It is worth noting that our observed low detection rate of the 108 marker loci and the detection of a single allele in 207 loci may be due to the germplasm pools that were not included in this study or validation set. Deeper testing of this panel on a broader range of sweetpotato germplasm is underway to see if these marker loci perform differently in extant germplasm. 

For this work, a high sequencing depth of ~200× was used by DArT to ensure the capture of rare alleles, which incurred an additional fee over the typical DArTag depth for diploids (50×). With more testing of the panel, it may be possible to reduce the cost per data point by reducing and optimizing the target sequencing depth at DArT and providing breeders with a better price point. Alternatively, breeders could choose to create a marker subset from this panel with loci that exhibit only biallelic markers to further reduce the sequencing depth and costs for some genomic applications [36].

### 4.2. Improving the DArTag Panel as a Global Community

A key benefit of DArTag over fixed array platforms is the ability to update and improve the panel as needed. The sweetpotato 3K DArTag panel is a pool of 3120 oligos, one for each locus, which is then used to generate the sequencing libraries from the assayed germplasm. Because the pool is created from individual oligo stocks, adding new loci or removing suboptimal loci can be easily achieved by creating a new oligo pool. For example, as new significant QTL markers and markers specific to other germplasm are detected, they can be targeted for inclusion in the original pool in the panel’s next version(s). Similarly, marker loci that are consistently poor-performing or consistently monomorphic are targets for locus removal from the panel in subsequent panel versions. DArT offers re-pooling services once per year at low or no cost, but more frequent requests could result in labor surcharges (Andrzej Kilian, personal communication). 

Breeding Insight (BI) at Cornell has organized a collaborative group of public breeders from the US, Jamaica, and the Caribbean, the CGIAR Potato Improvement Center (CIP), Costa Rica, and Taiwan to make updates to the 3K DArTag panel. Breeding Insight at Cornell is leading the working group to aggregate datasets and make recommendations to the group on loci to remove for poor performance (high missing, paralogous amplification, or excessive read depths) and loci that are truly monomorphic. The working group breeders are creating a list of SNP loci for inclusion in a new version of the panel, marking key QTL and loci for MAS. These efforts will culminate in the creation of v2.0 of the sweetpotato DArTag panel and are expected to be completed before the end of the 2024 calendar year.

One hurdle that flexible panels like DArTag face, that fixed panels and SNP chips do not, is a greater need for tracking marker set versions. Existing public data standards and resources do not adequately support the tracking of marker sets as they change over time as markers are removed while new ones are added. When updated iteratively, flexible marker panels can change more frequently and dramatically than fixed arrays. Ideally, each flexible marker panel version would be treated as a set and given a DOI or another standard indicator. The DOI or other standard indicator would allow for transparent disclosure of the markers in any panel version and associated marker metadata. The ability to track changes to the marker set will better abide by FAIR data principles. Currently, no community data standards or repositories can catalog and track changes in one version of a marker set to the next.

## 5. Conclusions

### Access to and Limitations of the Sweetpotato 3K DArTag Panel

This panel is publicly available and open for any researcher or breeder to order through DArT (https://www.diversityarrays.com, accessed on 31 October 2023). Panel users can follow the workflow we described from gDNA or tissue and proceed to genotypic data extraction in a three-to-four-week turnaround time. Several genotyping data reports can be requested, notably the raw data in FASTQ and the missing allele discovery file (MADC), which indicates the read depth of each microhaplotype in each sample. The ‘Allele_match_counts_collapsed’ contains read counts for reference and alternative alleles collapsed from all the microhaplotypes for each marker locus. The output of R package ‘updog’: ‘Allele_Dose_Report’ is also a part of the standard report. Other formats are available upon request. 

The panel and resulting data are suitable for marker-assisted selection, whole-genome association mapping, reconstruction of recombination patterns, allele dosage estimation, and parental confirmation in cultivated sweetpotato. The panel’s efficacy on breeding materials outside of North America and Jamaica is being tested but has not been verified. Researchers interested in initiating projects with DArT are encouraged to contact DArT directly for consultation.

We created a panel of 3K loci due to cost and technical reasons, but smaller complementary panels can be made at lower up-front and downstream usage costs. Adding a complementary 3K panel would nearly double the price of genotyping per sample but would result in more granular genotyping data.

## Data Availability

The FASTQ files from the whole-genome skim sequencing for the 47 sweetpotato accessions used for identifying the candidate SNP variants were deposited in the NCBI Short Read Archive under the BioProject ID PRJNA1022009. The code and data for the construction of the linkage map in MAPpoly are available in the GitHub repository for those interested in reproducing our analysis (https://github.com/mmollina/sweetpotato_mid_density_genotyping_platform, accessed on 15 April 2024).

## Supplementary Materials

The following supporting information can be downloaded at: https://www.mdpi.com/article/10.3390/genes15081047/s1. Table S1: Accessions used to construct and test the sweetpotato (*I. b.*) 3K DArTag panel. Germplasm accessions used whole genome sequencing and SNP database construction are indicated by Y in the “SNP discovery” column. Germplasm accessions used to validate the 3K panel are indicated by Y in the “Validation set” column. Table S2: Number of selected marker loci versus genes for each chromosome of the sweetpotato genome. Table S3: Number of microhaplotypes for the 3K DArTag panel from the validation project. Table S4: Missing data for samples of the validation set. Table S5: Missing data for markers of the validation set. File S1: Metadata associated with the 3K DArTag marker loci. File S2: MADC report of the validation set. Figure S1: Recombination fraction matrix for 60 F1 progeny from the Beauregard × Ruddy cross and the reciprocal cross. The graphic represents the pairwise recombination fraction matrix between markers positioned at both axes. In well-ordered linkage groups, we expect a gradient from hot colors in the diagonal (adjacent markers) to cold colors in the upper left and lower right corners, meaning that closer markers have a lower recombination (red) rate between them, and distant markers have a higher recombination rate (blue) between them. Figure S2: Sweetpotato genetic map construction. (A) Pipeline workflow for genetic map construction [34]. (B) Summary of the final map with 1092 unique markers. (C) Example haplotype reconstructions from MAPpoly1 for two individuals from the F1 population (BR1 and RB1). The x-axis represents the genetic map position, and the y-axis represents the probability of 0 to 1 within each of the six homologs shown as labels a, b, c, d from Beauregard (B) parent and e, f, g, h from Regal (R) parent across all linkage groups. The inversions in the probability of magnitudes between homologs from the same parent (different colors) represent possible regions of crossover occurrence.
