# Supplementary figures and images for: A Public Mid-Density Genotyping Platform for Hexaploid Sweetpotato (Ipomoea batatas [L.] Lam)
Source: Genes (Basel). 2024 Aug 9;15(8):1047. doi: 10.3390/genes15081047 (PMC11354173; doi:10.3390/genes15081047)

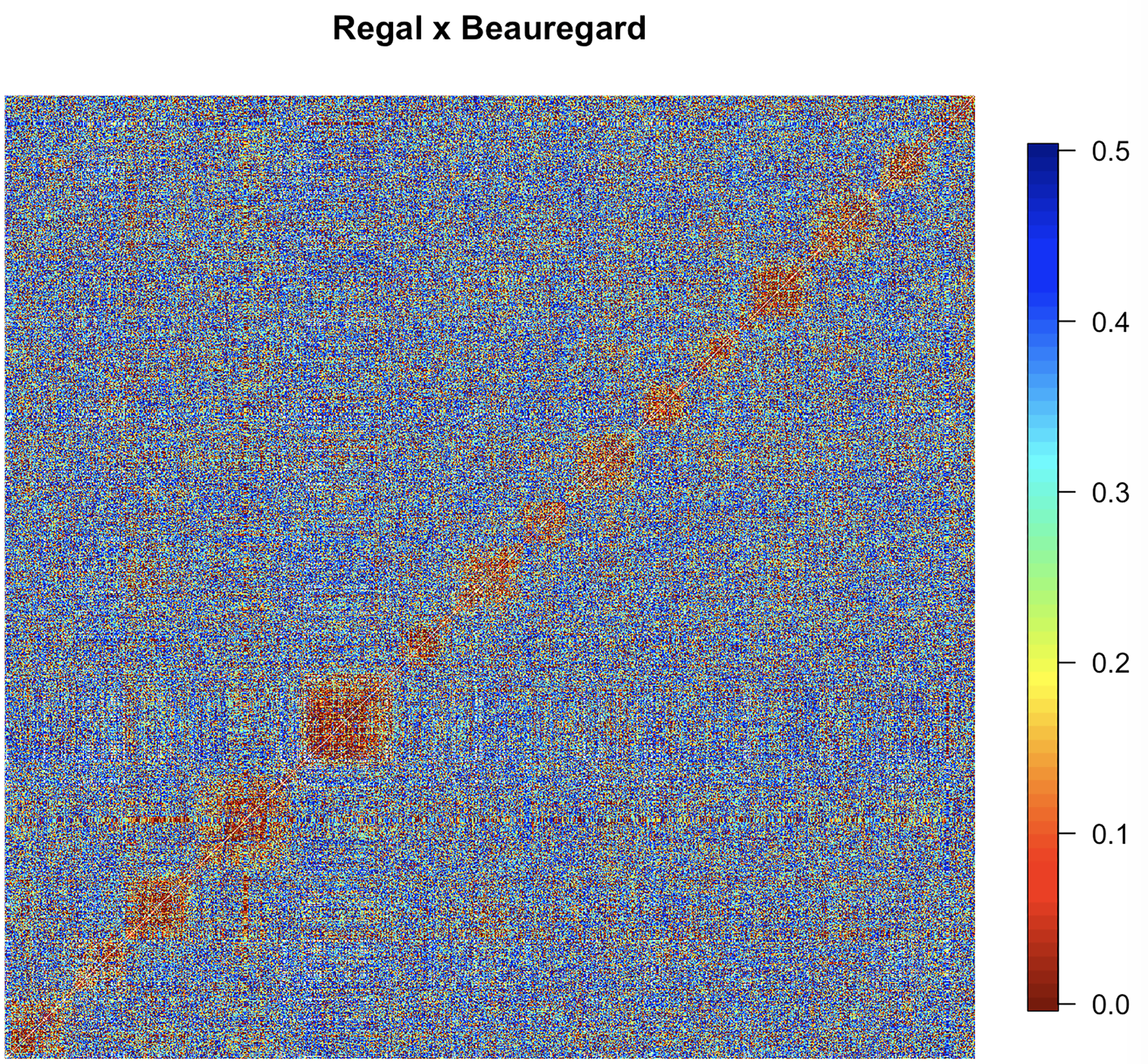

Supplement: Supplementary file 1 [file genes-15-01047-s001.zip › FigureS1_600dpi.png]
